# Adenocarcinoma with Mixed Subtypes in the Early and Advanced Gastric Cancer

**DOI:** 10.1155/2021/8497305

**Published:** 2021-10-29

**Authors:** Xixian Zhao, Yizhang Li, Zhenwei Yang, Hailin Zhang, Hongling Wang, Jun Lin, Jing Liu, Qiu Zhao

**Affiliations:** ^1^Department of Gastroenterology, Zhongnan Hospital of Wuhan University, Wuhan 430071, China; ^2^Hubei Clinical Center & Key Lab of Intestinal & Colorectal Diseases, Wuhan 430071, China

## Abstract

**Objective:**

Adenocarcinoma with mixed subtypes (AM) is a histological classification based on the WHO classification. We aimed to compare the prognosis among AM, classic adenocarcinoma (CA), mucinous adenocarcinoma (MAC), and signet-ring cell carcinoma (SRCC) in early and advanced gastric cancer (EGC and AGC), respectively.

**Methods:**

The Surveillance, Epidemiology, and End Results (SEER) database was queried from 2001 to 2016. Univariate and multivariate Cox analyses were performed to compare prognosis between AM and histologic subtypes of CA, SRCC, and MAC in ECG and ACG. A nomogram was established to predict the cancer-specific survival (CSS) of gastric cancer (GC) patients with AM. C-index, calibration curves, and receiver operating characteristic (ROC) and decision curve analysis (DCA) curves were applied to examine the accuracy and clinical benefits.

**Results:**

In the prognosis among these four histological subtypes in EGC patients, there are no differences. For AGC patients, AM had a significantly poorer prognosis compared with CA and MAC (*P*=0.003, 0.029) but similar prognosis to SRCC. A nomogram based on race, T stage, N stage, M stage, and surgical modalities was proposed to predict 1- and 3-year CSS for GC patients with AM (C-index: training cohort: 0.804, validation cohort: 0.748. 1- and 3-year CSS AUC: training cohort: 0.871 and 0.914, validation cohort: 0.810 and 0.798). 1- and 3-year CSS DCA curves showed good net benefits.

**Conclusions:**

EGC patients with AM had similar survival to those with CA, MAC, and SRCC. AM was an independent predictor of poor prognosis in AGC. A nomogram for predicting the prognosis of GC patients with AM was proposed to quantitatively assess the long-term survival.

## 1. Introduction

More than one million (1,033,701) new cases of gastric cancer (GC) were diagnosed globally in 2018, with 782,685 deaths [1]. 90% of gastric cancer is adenocarcinomas (ACs) [2]. There were multiple studies on the prognosis of GC patients with the histology of mucinous adenocarcinoma (MAC) and signet-ring cell carcinoma (SRCC) [3–7]. Clinical features and prognosis of SRCC were reported to be different between early gastric cancer and advanced gastric cancer (EGC and AGC) [8]. There may still be other histologic subtypes with distinct clinicopathologic characteristics and prognosis that need special concern in clinical management. Adenocarcinoma with mixed subtypes (AM) is a type of uncommon adenocarcinoma in GC based on WHO classification, which is defined as morphologically a combination of identifiable glandular (tubular/papillary) and signet-ring/poorly cohesive cellular components [9]. There were few researches on the prognosis of GC patients with AM, which only discussed EGC and included a small number of patients with AM [10, 11]. There is no report on the comparison of prognosis among AM, classic adenocarcinoma (CA), MAC, and SRCC in EGC and AGC, respectively.

In the present study, we compared the prognosis among EGC and AGC patients with AM and other histologic subtypes of CA, MAC, and SRCC based on Surveillance, Epidemiology, and End Results (SEER) database. In addition, we developed a predictive nomogram to quantify the survival estimates of GC patients with AM.

## 2. Material and Methods

### 2.1. Data Source and Patient Selection

A retrospective review of GC with histology of AM from the SEER database between 2001 and 2016 was performed. Data of GC (site recode ICD-O-3/WHO 2008 of “stomach”) patients with histology of AM (Code 8255), CA (Code 8144, 8210-8211, 8260–8263), MAC (Code 8480-8481), and SRCC (Code 8490) from 2004 to 2016 were collected from the SEER database. A total of 6679 patients were selected according to the following exclusion criteria: (1) GC was not the first diagnosed primary tumor. (2) The information of race, tumor size, the American Joint Committee on Cancer (AJCC) 7th Tumor Node Metastasis (TNM) stage, cause of death, and surgery was not available. (3) The time of follow-up was 0. EGC is defined as gastric cancer confined to the mucosa or submucosa regardless of the presence of lymph node metastasis (LNM). AGC is defined as gastric cancer exceeding the submucosa [8]. The baseline characteristics collected included sex, age, race, primary site, the AJCC 7th TNM stage, surgery, and tumor size. Follow-up data collected included overall survival (OS) and cancer-specific survival (CSS) and survival time.

This article does not contain any studies with human participants or animals performed by any of the authors. Informed consent is waived as SEER is a deidentified, publicly available cancer database.

### 2.2. Statistical Analysis

We firstly explored the frequency, incidence, and mortality of newly diagnosed GC patients with histology of AM. Rates were expressed as per 100,000 individuals and age-adjusted (2000 US Standard Population, 19 age groups). Annual percentage changes (APCs) were then calculated. The comparisons of clinicopathologic characteristics between AM and the other histologic subtypes including CA, MAC, and SRCC in EGC and AGC were performed with chi-square test. Univariate and multivariate Cox analyses were performed to explore the influence of different histologic subtypes on cancer-specific death. A multivariate Gray's competing risk regression model was then performed to adjust potential confounding factors.

Subsequently, the prognostic analysis was performed in GC patients with AM. Patients were assigned into training cohort (Alaska, Northern Plains, Pacific Coast, Southwest, *n* = 315) and validation cohort (East, *n* = 136) by geographic region of the United States. In the training cohort, univariate and multivariate Cox analyses were used to determine the independent prognostic factors. Significant prognostic factors were used to establish a nomogram to predict the 1- and 3-year CSS rates. Then, internal and external validations were performed. The validation cohorts were used for external validation. We used calibration curves and concordance index (C-index) curves to internally and externally evaluate the predictive accuracy of the nomogram (bootstraps with 500 resample). Receiver operating characteristic (ROC) curves were used for the internal and external validation of the nomogram for 1- and 3-year CSS rates. Decision curve analysis (DCA) was then performed to analyze the clinical usability of the nomogram for 1- and 3-year CSS rates.

IBM SPSS 23.0 and R software version 3.6 were utilized in performing all the above statistical analyses; and two‐tailed *P* < 0.05 indicated statistical significance.

## 3. Results

### 3.1. Incidence Trends

**Figure 1:**
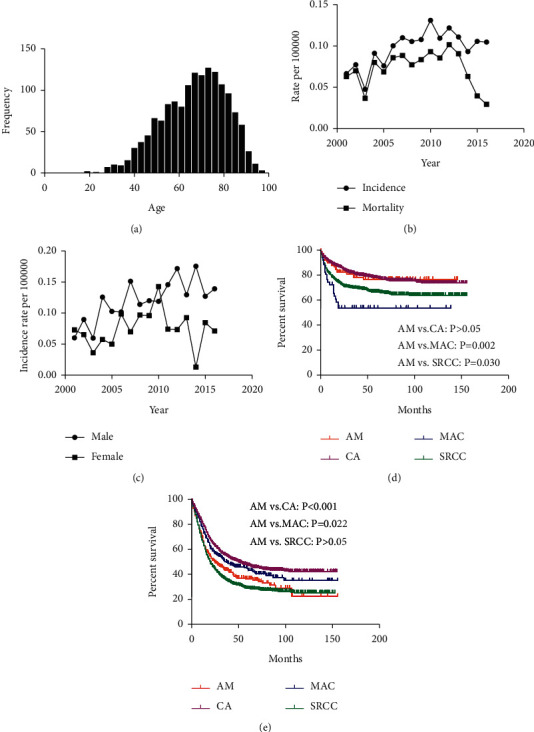
Incidence (a) of gastric cancer (GC) with adenocarcinoma with mixed subtypes (AM). The most common age at diagnosis was 72–74 years. Both the age-adjusted incidence rate and mortality rate of GC with AM (b). This increased trend (c) was similar regardless of sex. Comparisons of cancer-specific survival (d) in histological subtypes plotted with the Kaplan–Meier method in early gastric cancer (EGC). Comparisons of cancer-specific survival (e) in histological subtypes plotted with the Kaplan–Meier method in advanced gastric cancer (AGC).

The number of newly diagnosed GC patients with AM from 2001 to 2016 was divided by age, and the most common age at diagnosis was 72–74 years (Figure 1(a)). The trend from 2001 to 2016 in age‐adjusted incidence for GC patients with AM was illustrated with an APC of 3.7% (95% CI: 1.3–6.1) (Figure 1(b)). The mortality rate increased from 2001 to 2012 with an APC of 5.1% (95% CI: 1.1–9.2) but decreased from 2012 to 2016 with an APC of −28.1% (95% CI: −35.4–−20.1) (Figure 1(b)). In terms of genders, the incidence trend was quite different between the two groups (Figure 1(c)).

### 3.2. Comparisons of Clinicopathologic Differences between the Histology of AM and Other Histologic Subtypes in EGC and AGC Patients

In EGC patients, there were no significant differences in sex, tumor size, N stage, and M stage between AM and three other histologic subtypes. AM was more common in nonwhite patients than MAC and SRCC (Table 1, *P*=0.013, 0.033). MAC was more likely to be discovered in the body of stomach than AM (*P*=0.031). No significant difference in the distribution of age was observed between AM and MAC. AM patients are older than SRCC patients but younger than CA patients (*P*=0.025, <0.001).

In terms of AGC patients, there were no differences in the location of primary tumor between AM and SRCC. CA and MAC were found more frequently in the antrum and body of stomach, respectively (Table 2, *P*=0.002, <0.001). AM was more common in younger patients than CA. There was no significant difference in the distribution of sex, race, and M stage between AM and CA. However, AM was significantly associated with larger tumor sizes and advanced T stage and N stage, proving poor prognosis, compared with CA (*P*=0.018, <0.001, <0.001). As for the comparison of AM and MAC, no differences were found in age, race, tumor size, and M stage, while AM was more common in female, T4, and N2-3 stage patients (*P*=0.021, 0.046, 0.002). When comparing AM and SRCC, AM was significantly associated with male, nonwhite, larger tumor sizes, and the older (*P*=0.002, 0.015, 0.002, <0.001). On the contrary, SRCC was more likely to be found in patients with M1 stage compared with AM (*P*=0.009).

### 3.3. The Prognostic Value of Histologic Subtypes for OS and CSS in EGC and AGC Patients

1-year and 3-year OS rates for AM in EGC were 87.8% and 72.6%, respectively, and 1-year and 3-year CSS rates were 87.8% and 78.1%, respectively (Table 3). 1-year and 3-year OS rates for AM in AGC were 64.8% and 39.7%, respectively, and 1-year and 3-year CSS rates were 66.8% and 43.7%, respectively (Table 4). We firstly performed the Kaplan–Meier curves of four subtypes to investigate whether GC patients between AM and other histologic subtypes have different survival rates in EGC and AGC patients (Figure 1(d)). The log-rank test displayed that EGC patients with AM had similar prognosis to CA but were associated with better prognosis compared with MAC and SRCC (*P*=0.002, 0.030). However, in AGC patients, AM was associated with poorer prognosis compared with CA and MAC (Figure 1(e), *P* < 0.001, =0.022) but similar prognosis with SRCC.

**Figure 2:**
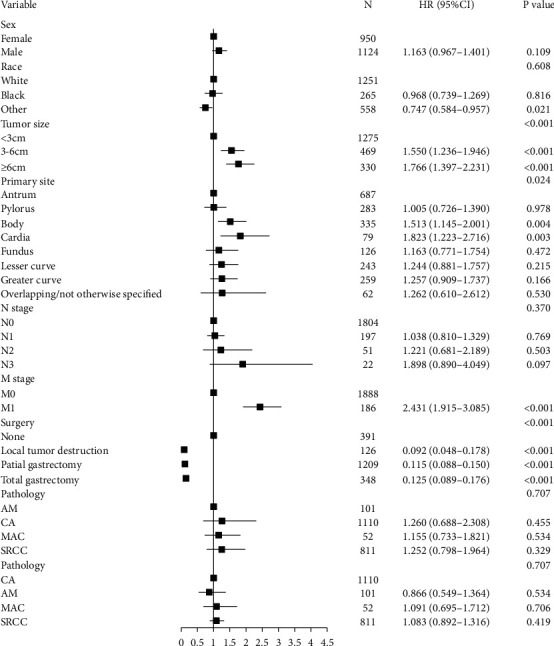
Forest plot showing results of the multivariate Cox regression model for the cancer-specific survival of histologic subtypes in early gastric cancer (EGC).

**Figure 3:**
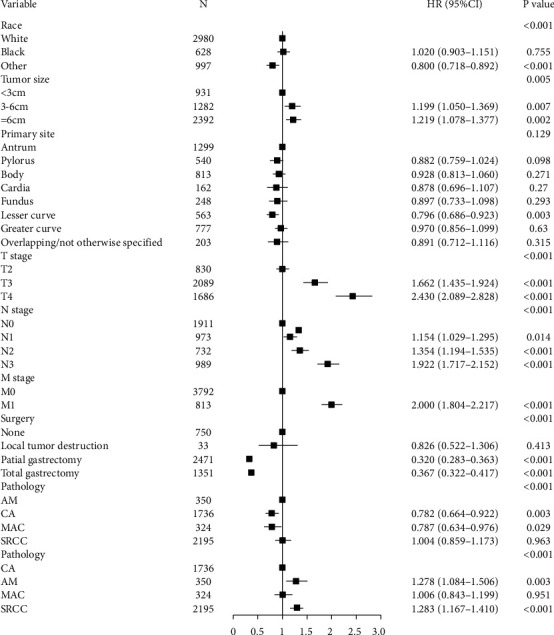
Forest plot showing results of the multivariate Cox regression model for the cancer-specific survival of histologic subtypes in advanced gastric cancer (AGC).

Multivariate Cox regression was performed for CSS to adjust potential confounding factors. The results revealed that the prognosis of four histologic subtypes had no difference in EGC patients (Figure 2). In AGC patients, AM had a significantly poorer prognosis compared with CA and MAC (Figure 3, HR: 0.782, 95% CI: 0.664–0.922, *P*=0.003; HR: 0.787, 95% CI: 0.634–0.976, *P*=0.029) but similar prognosis to SRCC. Taking death that is not related to GC into consideration, we also performed a multivariate Gray's competing risk regression model to adjust potential confounding factors, exhibiting similar results about the histologic subtypes (Tables 5 and 6).

### 3.4. Nomogram Predicting the Probability of CSS in GC Patients with AM and Validation

**Figure 4:**
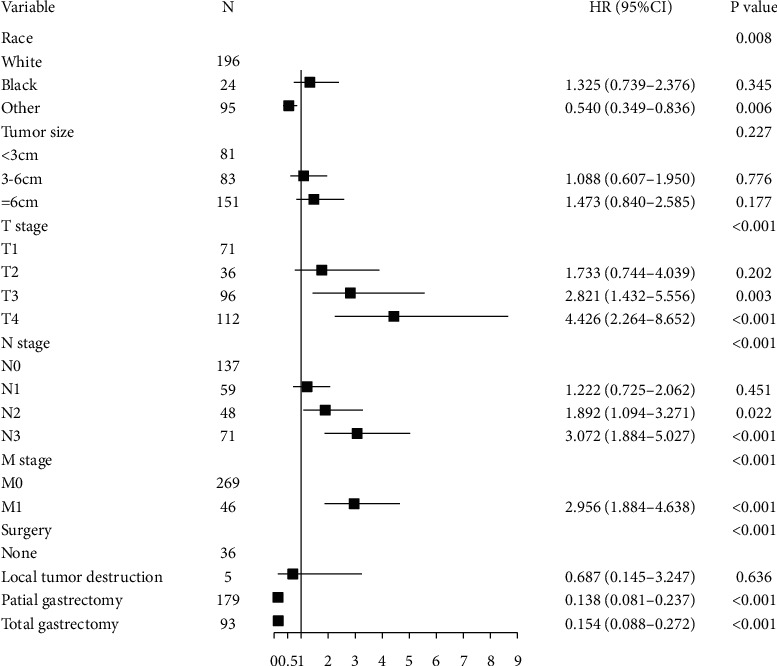
Forest plot showing results of the multivariate Cox regression model for the cancer-specific survival of gastric cancer patients with adenocarcinoma with mixed subtypes (AM).

We further explored the prognostic factors for CSS of GC patients with AM. In the training cohort, univariate and multivariate Cox regression analysis was performed. Race, T stage, N stage, M stage, and surgery were considered independent prognostic factors for the CSS in those patients with AM. In the multivariate Cox model, T3-4 stage, N2-3 stage, M1 stage, and no surgical modalities of partial gastrectomy and total gastrectomy were independently associated with the lower probability of CSS in GC patients with AM (Figure 4).

**Figure 5:**
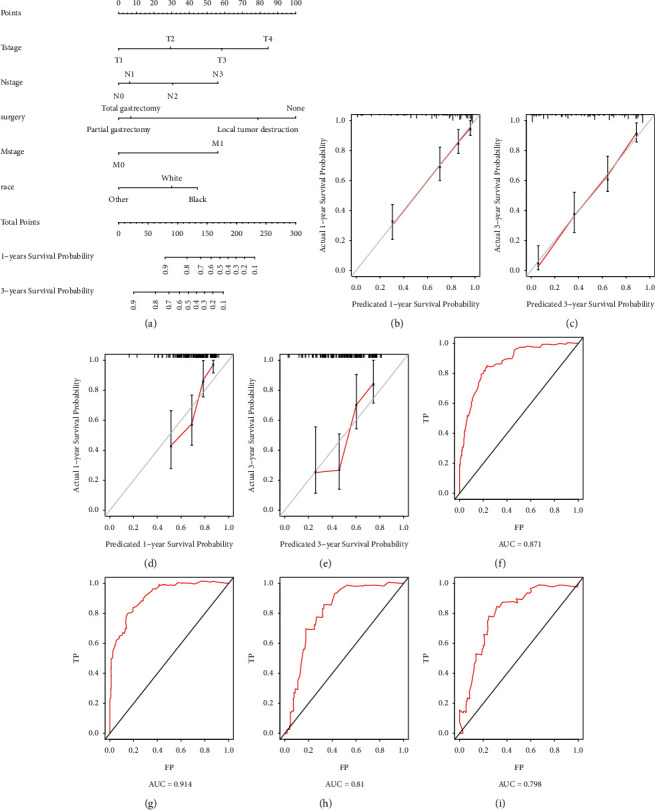
The nomogram (a) to predict the 1-year and 3-year cancer-specific survival (CSS) rates of GC patients with adenocarcinoma with mixed subtypes (AM). The calibration curve to verify the predictive ability for 1-year and 3-year CSS using variables from our developed nomogram in the training cohort (b, c) and validation cohort (d, e). The receiver operating characteristic (ROC) curve to verify the predictive ability for 1-year and 3-year CSS using variables from our developed nomogram in the training cohort (f, g) and validation cohort (h, i).

**Figure 6:**
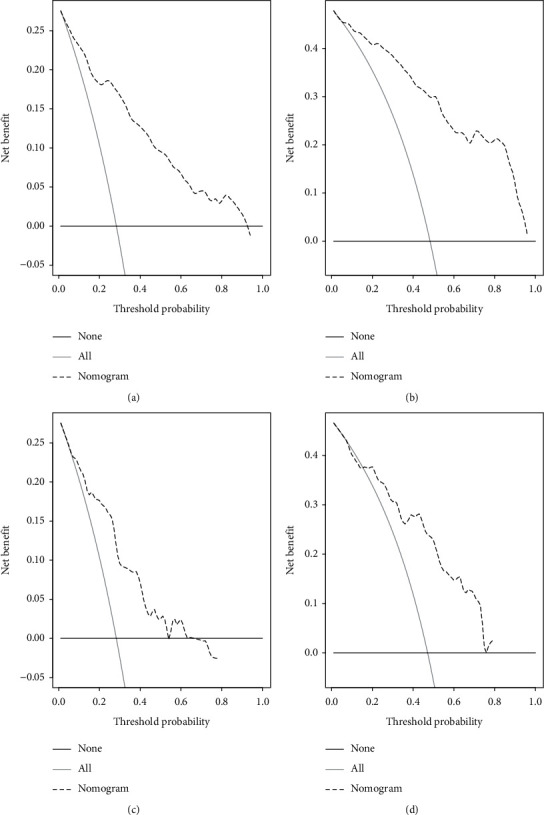
Decision curve analysis of nomogram for predicting 1-year and 3-year cancer-specific survival (CSS) in the training cohort (a, b) and validation cohort (c, d).

The CSS nomogram was constructed by incorporating those independent prognostic predictors (Figure 5(a)). The excellent C-index values (CSS in the training cohort: 0.804; CSS in the validation cohort: 0.748) and calibration curves of 1- and 3-year CSS in both internal and external validations indicated good agreements between the nomogram-predicted CSS probability and actual CSS probability (Figures 5(b)–5(e)). We further adopted ROC curves for the internal and external validations of the nomogram (Figures 5(f)–5(i)). The favorable 1- and 3-year AUC values (training cohort: AUC = 0.871 and 0.914; validation cohort: AUC = 0.810 and 0.798) illustrated good ability for CSS prediction in GC patients with AM. In addition, the nomogram's 1- and 3-year DCA curves demonstrated good net benefits across a range of death risks in both the training cohort and validation cohort (Figure 6).

## 4. Discussion

In this study, the incidence of GC with AM steadily increased over the past 16 years to approximately 0.1 per 100,000. To our knowledge, there are few studies on the incidence of GC with AM. The mortality of GC with AM increased from 2001 to 2012 but decreased from 2012 to 2016. GC with AM was more frequently discovered in men; and the male incidence showed a steady increase trend, while the female incidence kept a trend of fluctuation.

Our conclusions about the prognosis of MAC were mainly in consistency with previous reports [12–14]. The prognosis of MAC had no difference with CA in both EGC and AGC. In terms of SRCC, SRCC had comparable prognosis with CA in EGC but poorer prognosis in AGC. Though it is widely accepted that SRCC is an independent predictor of poor prognosis in advanced gastric adenocarcinomas (GA), the prognosis of SRCC in EGC remains highly controversial [8, 15–18]. In our study, the comparison object of SRCC was CA that was different from previous studies. The reference value of previous researches for our study is questionable.

There were some studies about the clinical characteristics and prognosis of mixed-type EGC based on Lauren's classification [19–21]. According to the WHO classification, the mixed carcinoma based on Lauren's classification is different from AM.

Our study found that the clinicopathologic features between adenocarcinoma with mixed subtypes and other histologic types, including classical adenocarcinoma, mucinous adenocarcinoma, and signet-ring cell carcinoma, were different in EGC and AGC. We found that there were no significant differences in tumor size, N stage, and M stage between AM and three other histologic subtypes in EGC. However, we observed a larger tumor size in AM compared with CA and SRCC, a higher T stage, and a higher N stage in AM compared with CA and MAC in AGC. Our study revealed that AM did not have relatively poor clinicopathological features in EGC, which is not consistent with previous researches [11]. We think that this is due to the fact that the comparison is not just with adenocarcinoma in the research mentioned above. The number of patients with AM in this research was small. In EGC, we found that AM had a comparable prognosis with CA, MAC, and SRCC. To our knowledge, AM was not well documented in the literature of AGC. In AGC, AM was significantly associated with more aggressive clinicopathological features compared with CA, MAC, and SRCC. AGC patients with AM had a poorer prognosis compared with CA and MAC but a comparable prognosis with SRCC. AM was an independent risk factor for a poor outcome in AGC. We also collected the 1-year and 3-year survival rates of AM in EGC and AGC, which was the first study of survival data of AM in GC.

Furthermore, we explored the independent prognostic predictor of GC with AM. In our study, T3-4 stage, N2-3 stage, M1 stage, and no surgical modalities of partial gastrectomy and total gastrectomy were proposed to be independent predictors of poor CSS of GC patients with AM. Our study was the first study about the predictive model for prognostic assessment in GC with AM. We believe that the establishment of our prognostic scoring system based on GC patients with AM is of clinical significance. We evaluated the value of this nomogram to predict 1- and 3-year CSS rates of GC patients with AM by C-index, calibration curves, and ROC curves, which displayed good agreements in both training cohort and validation cohort. Furthermore, DCA demonstrated the benefits and clinical utility of the predictive power of our nomogram.

This study has some limitations. Firstly, this is a retrospective analysis of patients using SEER database. Secondly, we were unable to analyze the disease-free survival of patients and obtain data regarding radiotherapy, chemotherapy, and targeted therapies received in localized and advanced disease due to limitations of the database. Thirdly, we did not discuss the effect of differentiation on prognosis. Fourthly, selection bias might exist after the case screening. Besides, there was no information of their genotypes, genetic data among these histologic subtypes that may be the prognosis factors, such as human epidermal-growth-factor receptor 2 (HER-2), microsatellite instable (MSI), and Epstein–Barr Virus (EBV) [22–24].

## 5. Conclusions

In summary, there were no differences in the survival among those EGC patients with AM, CA, MAC, and SRCC. AGC patients with AM had poorer prognosis than those with CA and MAC but similar prognosis to those with SRCC. In addition, we established a nomogram to quantify the CSS of GC patients with AM to help doctors predict the survival of these patients, so as to determine the treatment strategies in actual clinical practice.

## Figures and Tables

**Table 1:** Comparisons of the clinicopathologic features between adenocarcinoma with mixed subtypes and other histologic types, including classical adenocarcinoma, mucinous adenocarcinoma, and signet-ring cell carcinoma in early gastric cancer.

Variable	AM^a^ (%) *N* = 101	CA^b^ (%) *N* = 1110	MAC^c^ (%) *N* = 52	SRCC^d^ (%) *N* = 811	*P* value	AM vs. CA, *P* value	AM vs. MAC, *P* value	AM vs. SRCC, *P* value
Sex								
Female	47 (46.5%)	458 (42.3%)	22 (41.3%)	423 (52.2%)	<0.001	0.303	0.619	0.286
Male	54 (53.5%)	652 (57.7%)	30 (58.7%)	388 (47.8%)				
Age								
<65	42 (41.6%)	263 (23.7%)	19 (36.5%)	433 (53.4%)	<0.001	<0.001	0.546	0.025
≥65	59 (58.4%)	847 (76.3%)	33 (63.5%)	378 (46.6%)				
Race								
White	53 (52.5%)	631 (56.8%)	40 (76.9%)	527 (65.0%)	0.001	0.675	0.013	0.033
Black	14 (13.9%)	148 (13.3%)	4 (8.0%)	99 (12.2%)				
Others	34 (33.7%)	331 (29.8%)	8 (15.4%)	185 (22.8%)				
Tumor size								
<3 cm	59 (58.4%)	689 (62.1%)	20 (38.5%)	507 (62.5%)	0.018	0.765	0.057	0.507
3–6 cm	26 (25.7%)	257 (23.2%)	18 (34.6%)	168 (20.7%)				
≥6 cm	16 (15.8%)	164 (14.8%)	14 (26.9%)	136 (16.8%)				
Primary site								
Antrum	27 (28.2%)	418 (26.3%)	14 (26.9%)	228 (25.9%)	<0.001	0.148	0.031	0.589
Pylorus	18 (13.3%)	146 (7.2%)	3 (5.8%)	116 (12.7%)				
Body	20 (18.0%)	158 (37.8%)	24 (46.2%)	133 (17.9%)				
Cardia	4 (3.1%)	46(2.9%)	2 (3.8%)	27 (2.8%)				
Fundus	7 (6.0%)	60 (4.0%)	1 (1.9%)	58 (6.4%)				
Lesser curve	8 (9.1%)	126 (6.7%)	2 (3.8%)	107 (12.8%)				
Greater curve	16 (17.7%)	124 (13.3%)	6 (11.5%)	113 (17.6%)				
Overlapping/not otherwise specified	1 (4.7%)	32 (1.9%)	0 (0%)	29 (4.1%)				
N stage								
N0	82 (81.1%)	992 (89.4%)	41 (78.8%)	689 (85.0%)	0.017	0.053	0.672	0.597
N1	13 (12.9%)	90 (8.1%)	6 (11.5%)	88 (10.9%)				
N2	5 (5.0%)	20 (1.8%)	3 (5.8%)	23 (2.8%)				
N3	1 (0.9%)	8 (0.7%)	2 (3.8%)	11 (13.6%)				
M stage								
M0	91 (85.6%)	1050 (94.6%)	43 (82.7%)	704 (86.8%)	<0.001	0.064	0.188	0.351
M1	10 (14.4%)	60 (5.4%)	9 (17.3%)	107 (13.2%)				
Surgery								
None	17 (16.8%)	134 (12.1%)	19 (36.5%)	221 (27.3%)	<0.001	0.038	0.043	0.008
Local tumor destruction	5 (5.0%)	110 (9.9%)	1 (1.9%)	10 (1.2%)				
Partial gastrectomy	57 (56.4%)	708 (63.8%)	25 (48.1%)	419 51.7%)				
Total gastrectomy	22 (21.8%)	158 (14.2%)	7 (13.5%)	161 (19.9%)				

^a^Adenocarcinoma with mixed subtypes; ^b^classical adenocarcinoma; ^c^mucinous adenocarcinoma; ^d^signet-ring cell carcinoma.

**Table 2:** Comparisons of the clinicopathologic features between adenocarcinoma with mixed subtypes and other histologic types, including classical adenocarcinoma, mucinous adenocarcinoma, and signet-ring cell carcinoma in advanced gastric cancer.

Variable	AM^a^ (%) *N* = 350	CA^b^ (%) *N* = 1736	MAC^c^ (%) *N* = 324	SRCC^d^ (%) *N* = 2195	*P* value	AM vs. CA, *P* value	AM vs. MAC, *P* value	AM vs. SRCC, *P* value
Sex								
Female	132 (37.7%)	639 (36.8%)	95 (29.3%)	1025 (46.7%)	<0.001	0.749	0.021	0.002
Male	218 (62.3%)	1097 (63.2%)	229 (70.7%)	1170 (53.3%)				
Age								
<65	155 (44.3%)	533 (30.7%)	134 (41.4%)	1270 (57.9%)	<0.001	<0.001	0.443	<0.001
≥65	195 (55.7%)	1203 (69.3%)	190 (58.6%)	925 (42.1%)				
Race								
White	221 (63.1%)	1027 (59.2%)	229 (70.7%)	1503 (68.5%)	<0.001	0.355	0.080	0.015
Black	53 (15.1%)	278 (16.0%)	45 (13.9%)	252 (11.5%)				
Others	76 (21.7%)	431 (24.8%)	50 (15.4%)	440 (20.0%)				
Tumor size								
<3 cm	54 (15.4%)	321 (18.5%)	48 (14.8%)	508 (23.1%)	<0.001	0.018	0.787	0.002
3–6 cm	89 (25.4%)	532 (30.6%)	90 (27.8%)	571 (26.0%)				
≥6 cm	207 (59.1%)	883 (50.9%)	186 (57.4%)	1116 (50.8%)				
Primary site								
Antrum	100 (28.6%)	564 (32.5%)	85 (26.2%)	550 (25.1%)	<0.001	0.038	<0.001	0.543
Pylorus	42 (12.0%)	208 (12.0%)	24 (7.4%)	266 (12.1%)				
Body	61 (17.4%)	230 (13.2%)	118 (36.4%)	404 (18.4%)				
Cardia	10 (2.9%)	87 (5.0%)	9 (2.8%)	56 (2.6%)				
Fundus	20 (5.7%)	83 (4.8%)	14 (4.3%)	131 (6.0%)				
Lesser curve	33 (9.4%)	228 (13.1%)	23 (7.1%)	279 (12.7%)				
Greater curve	64 (18.3%)	253 (14.6%)	44 (13.6%)	416 (19.0%)				
Overlapping/not otherwise specified	20 (5.7%)	83 (4.8%)	7 (2.2%)	93 (4.2%)				
T stage								
T2	52 (14.9%)	379 (21.8%)	58 (17.9%)	341 (15.5%)	<0.001	<0.001	0.046	0.931
T3	149 (42.6%)	866 (49.9%)	158 (48.8%)	916 (41.7%)				
T4	149 (42.6%)	491 (28.3%)	108 (33.3%)	938 (42.7%)				
N stage								
N0	113 (32.3%)	806 (46.4%)	138 (42.6%)	854 (38.9%)	<0.001	<0.001	0.002	0.063
N1	70 (2.0%)	379 (21.8%)	77 (23.8%)	447 (20.4%)				
N2	67 (19.1%)	274 (15.8%)	50 (15.4%)	341 (15.5%)				
N3	100 (28.6%)	277 (16.0%)	59 (18.2%)	553 (25.2%)				
M stage								
M0	295 (84.3%)	1510 (87.0%)	272 (84.0%)	1715 (78.1%)	<0.001	0.178	0.905	0.009
M1	55 (15.7%)	226 (13.0%)	52 (16.0%)	480 (21.9%)				
Surgery								
None	43 (12.3%)	175 (10.1%)	44 (13.6%)	488 (22.2%)	<0.001	0.280	0.458	<0.001
Local tumor destruction	2 (0.6%)	11 (0.6%)	4 (1.2%)	16 (0.7%)				
Partial gastrectomy	200 (57.1%)	1085 (62.5%)	194 (59.9%)	992 (45.2%)				
Total gastrectomy	105 (30.0%)	465 (26.8%)	82 (25.3%)	699 (31.8%)				

^a^Adenocarcinoma with mixed subtypes; ^b^classical adenocarcinoma; ^c^mucinous adenocarcinoma; ^d^signet-ring cell carcinoma.

**Table 3:** 1-year and 3-year overall survival (OS) rates and cancer-specific survival (CSS) rates of patients with 4 histological subtypes (adenocarcinoma with mixed subtypes, classical adenocarcinoma, mucinous adenocarcinoma, and signet-ring cell carcinoma) in early gastric cancer.

Pathological characteristics	1-year OS^e^ rate (%)	3-year OS^e^ rate (%)	1-year CSS^f^ rate (%)	3-year CSS_f_ rate (%)
AM^a^	87.8 (81.3 − 94.3)	72.6 (63.3 − 81.9)	87.8 (81.3 − 94.3)	78.1 (69.6 − 86.7)
CA^b^	83.9 (81.7 − 86.1)	70.9 (68.1 − 73.8)	89.7 (87.8 − 91.5)	81.3 (78.8 − 83.8)
MAC^c^	68.5 (55.8 − 81.3)	44.0 (29.7 − 58.3)	72.4 (60.1 − 84.8)	53.8 (39.4 − 68.2)
SRCC^d^	75.6 (72.5 − 78.6)	64.8 (61.3 − 68.3)	78.6 (75.6 − 81.5)	69.6 (66.2 − 73.0)

^a^Adenocarcinoma with mixed subtypes; ^b^classical adenocarcinoma; ^c^mucinous adenocarcinoma; ^d^signet-ring cell carcinoma; ^e^overall survival; ^f^cancer-specific survival.

**Table 4:** 1-year and 3-year overall survival (OS) rates and cancer-specific survival (CSS) rates of patients with 4 histological subtypes (adenocarcinoma with mixed subtypes, classical adenocarcinoma, mucinous adenocarcinoma, and signet-ring cell carcinoma) in advanced gastric cancer.

Pathological characteristics	1-year OS^e^ rate (%)	3-year OS^e^ rate (%)	1-year CSS^f^ rate (%)	3-year CSS^f^ rate (%)
AM^a^	64.8 (59.5 − 70.0)	39.7 (33.9 − 45.5)	66.8 (61.5 − 72.0)	43.7 (37.7 − 49.7)
CA^b^	76.2 (74.1 − 78.3)	49.9 (47.3 − 52.5)	80.3 (78.4 − 82.3)	55.5 (52.9 − 58.2)
MAC^c^	73.7 (68.8 − 78.6)	44.9 (39.0 − 50.8)	76.3 (71.5 − 81.0)	50.5 (44.4 − 56.6)
SRCC^d^	63.8 (61.7 − 65.9)	33.6 (31.4 − 35.8)	65.7 (63.7 − 67.8)	36.4 (34.1 − 38.7)

^a^Adenocarcinoma with mixed subtypes; ^b^classical adenocarcinoma; ^c^mucinous adenocarcinoma; ^d^signet-ring cell carcinoma; ^e^overall survival; ^f^cancer-specific survival.

**Table 5:** Results of competing risks regression with inclusion of possible risk factors in patients with early gastric cancer.

Variable	Subdistribution hazard ratio	*P* value
Sex		
Female	1.218 (1.014 − 1.463)	0.035
Male		
Age		
<65	1 (reference)	
≥65	1.431 (1.177 − 1.739)	<0.001
Race		
White	1 (reference)	
Black	0.985 (0.750 − 1.294)	0.910
Others	0.774 (0.614 − 0.975)	0.029
Tumor size		
<3 cm	1 (Reference)	
3–6 cm	1.518 (1.213 − 1.900)	<0.001
≥6 cm	1.692 (1.342 − 2.134)	<0.001
Primary site		
Antrum	1 (reference)	
Pylorus	1.027 (0.761 − 1.384)	0.860
Body	1.539 (1.169 − 2.026)	0.002
Cardia	2.008 (1.360 − 2.964)	<0.001
Fundus	1.165 (0.809 − 1.680)	0.410
Lesser curve	1.173 (0.799 − 1.722)	0.410
Greater curve	1.306 (0.962 − 1.771)	0.087
Overlapping/not otherwise specified	1.086 (0.506 − 2.331)	0.830
N stage		
N0	1 (reference)	
N1	1.033 (0.803 − 1.330)	0.800
N2	1.234 (0.672 − 2.266)	0.500
N3	1.882 (1.073 − 3.301)	0.027
M stage		
M0	1 (reference)	
M1	3.184 (2.479 − 4.090)	<0.001
Surgery		
None	1 (reference)	
Local tumor destruction	0.112 (0.059 − 0.213)	<0.001
Partial gastrectomy	0.156 (0.120 − 0.205)	<0.001
Total gastrectomy	0.174 (0.123 − 0.245)	<0.001
Pathology		
AM^a^	1 (reference)	
CA^b^	1.035 (0.666 − 1.608)	0.520
MAC^c^	1.794 (0.697 − 2.045)	0.880
SRCC^d^	1.289 (0.840 − 1.976)	0.240

^a^Adenocarcinoma with mixed subtypes; ^b^classical adenocarcinoma; ^c^mucinous adenocarcinoma; ^d^signet-ring cell carcinoma.

**Table 6:** Results of competing risks regression with inclusion of possible risk factors in patients with advanced gastric cancer.

Variable	Subdistribution hazard ratio	*P* value
Sex		
Female	1 (reference)	
Male	1.035 (0.950 − 1.129)	0.430
Age		
<65	1 (reference)	
≥65	1.307 (1.198 − 1.427)	<0.001
Race		
White	1 (reference)	
Black	1.010 (0.887 − 1.149)	0.880
Others	0.804 (0.722 − 0.895)	<0.001
Tumor size		
<3 cm	1 (reference)	
3–6 cm	1.189 (1.041 − 1.359)	0.011
≥6 cm	1.235 (1.090 − 1.399)	<0.001
Primary site		
Antrum	1 (reference)	
Pylorus	0.839 (0.720 − 0.977)	0.024
Body	0.930 (0.807 − 1.072)	0.320
Cardia	0.896 (0.713 − 1.126)	0.350
Fundus	0.841 (0.689 − 1.029)	0.093
Lesser curve	0.788 (0.677 − 0.916)	0.002
Greater curve	0.969 (0.858 − 1.095)	0.620
Overlapping/not otherwise specified	0.856 (0.696 − 1.053)	0.140
T stage		
T2	1 (reference)	
T3	1.687 (1.462 − 1.947)	<0.001
T4	2.350 (2.022 − 2.732)	<0.001
N stage		
N0	1 (reference)	
N1	1.156 (1.028 − 1.300)	0.016
N2	1.403 (1.231 − 1.599)	<0.001
N3	1.889 (1.679 − 2.126)	<0.001
M stage		
M0	1 (reference)	
M1	2.094 (1.878 − 2.334)	<0.001
Surgery		
None	1 (reference)	
Local tumor destruction	0.844 (0.537 − 1.327)	0.460
Partial gastrectomy	0.347 (0.304 − 0.397)	<0.001
Total gastrectomy	0.409 (0.357 − 0.469)	<0.001
Pathology		
AM^a^	1 (reference)	
CA^b^	0.769 (0.611 − 0.967)	<0.001
MAC^c^	0.729 (0.611 − 0.869)	0.025
SRCC^d^	1.045 (0.879 − 1.243)	0.620

^a^Adenocarcinoma with mixed subtypes; ^b^classical adenocarcinoma; ^c^mucinous adenocarcinoma; ^d^signet-ring cell carcinoma.

## Data Availability

The data used to support the findings of this study are included within the article.

## Abbreviations

ACs: Adenocarcinomas
AGC: Advanced gastric cancer
AJCC: American Joint Committee on Cancer
AM: Adenocarcinoma with mixed subtypes
APCs: Annual percentage changes
CA: Classic adenocarcinoma
C-index: Concordance index
CSS: Cancer-specific survival
DCA: Decision curve analysis
EBV: Epstein–Barr virus
EGC: Early gastric cancer
GC: Gastric cancer
HER-2: Human epidermal-growth-factor receptor 2
LNM: Lymph node metastasis
MAC: Mucinous adenocarcinoma
MSI: Microsatellite instable
OS: Overall survival
ROC: Receiver operating characteristic
SEER: Surveillance, Epidemiology, and End Results
SRCC: Signet-ring cell carcinoma
TNM: Tumor node metastasis.

## References

[1] Bray F., Ferlay J., Soerjomataram I., Siegel R. L., Torre L. A., Jemal A. (2018). Global cancer statistics 2018: GLOBOCAN estimates of incidence and mortality worldwide for 36 cancers in 185 countries. *CA: A Cancer Journal for Clinicians*.

[2] Smyth E. C., Verheij M., Allum W., Cunningham D., Cervantes A., Arnold D. (2016). Gastric cancer: ESMO Clinical Practice Guidelines for diagnosis, treatment and follow-up. *Annals of Oncology*.

[3] Lee H. H., Song K. Y., Park C. H., Jeon H. M. (2012). Undifferentiated-type gastric adenocarcinoma: prognostic impact of three histological types. *World Journal of Surgical Oncology*.

[4] Kawamura H., Kondo Y., Osawa S. (2001). A clinicopathologic study of mucinous adenocarcinoma of the stomach. *Gastric Cancer*.

[5] de Aguiar V. G., Segatelli V., Macedo A. L. d. V. (2019). Signet ring cell component, not the Lauren subtype, predicts poor survival: an analysis of 198 cases of gastric cancer. *Future Oncology*.

[6] Pernot S., Voron T., Perkins G., Lagorce-Pages C., Berger A., Taieb J. (2015). Signet-ring cell carcinoma of the stomach: impact on prognosis and specific therapeutic challenge. *World Journal of Gastroenterology*.

[7] Chu Y.-N., Yu Y.-N., Jing X. (2019). Feasibility of endoscopic treatment and predictors of lymph node metastasis in early gastric cancer. *World Journal of Gastroenterology*.

[8] Kao Y.-C., Fang W.-L., Wang R.-F. (2019). Clinicopathological differences in signet ring cell adenocarcinoma between early and advanced gastric cancer. *Gastric Cancer*.

[9] Bosman F. T., Carneiro F., Hruban R. H., Theise N. D. (2010). *WHO Classification of Tumours of the Digestive System*.

[10] Han J. P., Hong S. J., Kim H. K. (2015). Long-term outcomes of early gastric cancer diagnosed as mixed adenocarcinoma after endoscopic submucosal dissection. *Journal of Gastroenterology and Hepatology*.

[11] Du M., Chen L., Cheng Y. (2019). Tumor budding and other risk factors of lymph node metastasis in submucosal early gastric carcinoma. *The American Journal of Surgical Pathology*.

[12] Cai L., Li Y., Yang X.-W. (2018). Prognostic significance of mucinous component in gastric adenocarcinoma after radical D2 gastrectomy. *OncoTargets and Therapy*.

[13] Yin C., Li D., Sun Z. (2012). Clinicopathologic features and prognosis analysis of mucinous gastric carcinoma. *Medical Oncology*.

[14] Liu K., Wan J., Bei Y., Chen X., Lu M. (2017). Prognostic impact of different histological types on gastric adenocarcinoma: a surveillance, epidemiology, and end results database analysis. *Pathology and Oncology Research*.

[15] Kwon K.-J., Shim K.-N., Song E.-M. (2014). Clinicopathological characteristics and prognosis of signet ring cell carcinoma of the stomach. *Gastric Cancer*.

[16] Kim B. S., Oh S. T., Yook J. H., Kim B. S. (2014). Signet ring cell type and other histologic types: differing clinical course and prognosis in T1 gastric cancer. *Surgery*.

[17] Lee S. H., Jee S. R., Kim J. H., Seol S. Y. (2015). Intramucosal gastric cancer. *European Journal of Gastroenterology and Hepatology*.

[18] Chon H. J., Hyung W. J., Kim C. (2017). Differential prognostic implications of gastric signet ring cell carcinoma. *Annals of Surgery*.

[19] Chen Y.-C., Fang W.-L., Wang R.-F. (2016). Clinicopathological variation of lauren classification in gastric cancer. *Pathology and Oncology Research*.

[20] Hwang C.-S., Ahn S., Lee B.-E. (2016). Risk of lymph node metastasis in mixed-type early gastric cancer determined by the extent of the poorly differentiated component. *World Journal of Gastroenterology*.

[21] Pyo J. H., Lee H., Min B.-H. (2017). Early gastric cancer with a mixed-type Lauren classification is more aggressive and exhibits greater lymph node metastasis. *Journal of Gastroenterology*.

[22] Elimova E., Wadhwa R., Shiozaki H. (2015). Molecular biomarkers in gastric cancer. *Journal of the National Comprehensive Cancer Network*.

[23] Yang G., Zheng R.-Y., Jin Z.-S. (2019). Correlations between microsatellite instability and the biological behaviour of tumours. *Journal of Cancer Research and Clinical Oncology*.

[24] Nishikawa J., Iizasa H., Yoshiyama H. (2018). Clinical importance of Epstein⁻Barr virus-associated gastric cancer. *Cancers*.

[25] Zhao X., Li Y., Yang Z. (2021). Adenocarcinoma with mixed subtypes in the early and advanced gastric cancer. *Research Square*.

